# Chemically imaging bacteria with super-resolution SERS on ultra-thin silver substrates

**DOI:** 10.1038/s41598-017-08915-w

**Published:** 2017-08-22

**Authors:** Aeli P. Olson, Kelsey B. Spies, Anna C. Browning, Paula A. G. Soneral, Nathan C. Lindquist

**Affiliations:** 1 Physics Department, Bethel University, St Paul, MN 55112 USA; 2 Biology Department, Bethel University, St Paul, MN 55112 USA

## Abstract

Plasmonic hotspots generate a blinking Surface Enhanced Raman Spectroscopy (SERS) effect that can be processed using Stochastic Optical Reconstruction Microscopy (STORM) algorithms for super-resolved imaging. Furthermore, by imaging through a diffraction grating, STORM algorithms can be modified to extract a full SERS spectrum, thereby capturing spectral as well as spatial content simultaneously. Here we demonstrate SERS and STORM combined in this way for super-resolved chemical imaging using an ultra-thin silver substrate. Images of gram-positive and gram-negative bacteria taken with this technique show excellent agreement with scanning electron microscope images, high spatial resolution at <50 nm, and spectral SERS content that can be correlated to different regions. This may be used to identify unique chemical signatures of various cells. Finally, because we image through as-deposited, ultra-thin silver films, this technique requires no nanofabrication beyond a single deposition and looks at the cell samples from below. This allows direct imaging of the cell/substrate interface of thick specimens or imaging samples in turbid or opaque liquids since the optical path doesn’t pass through the sample. These results show promise that super-resolution chemical imaging may be used to differentiate chemical signatures from cells and could be applied to other biological structures of interest.

## Introduction

Optical microscopy has historically been diffraction limited as defined by Abbe’s limit, with resolution based upon the numerical aperture of the imaging system and the wavelength of the light. The smallest wavelength of visible light for non-destructive imaging of living or delicate samples gives an optical resolution limit on the order of 200 nm. Super-resolution imaging techniques continue to be developed in order to extend the boundaries and capabilities of optical microscopy past diffraction-limited resolution limits^1–4^. These techniques are varied in their approaches, but many include the addition of fluorescent tags and labels into the system. Even one of the most straightforward options, structured illumination microscopy^5^, requires the use of fluorescent samples^6^. Therefore, the option of super-resolution imaging without the use of fluorescent tags is valuable as the introduction of another substance into the system introduces extra complexity and may alter the natural state of the sample. Research into high-resolution label-free imaging is being explored in a variety of ways including using a pump-probe process^7^ or variations on holographic imaging^8^ and coherent scattering^9^. Alternative techniques exploit the optical properties of nanostructures as designer imaging substrates^10, 11^. In particular, the field of plasmonics^12–14^ exploits resonances in metallic nanostructures and is an approach to optical nanoscopy that has gathered significant attention^15^. Plasmons are oscillations of the free electrons in metallic nanostructures that can generate intense local fields, sometimes called “hotspots,” that are on the order of 10 nm in size. Plasmonic hotspots and surface plasmon resonance have been used for many applications, including biosensing^16–19^, Surface Enhanced Raman Spectroscopy (SERS)^20–24^, tip-enhanced imaging^25^, and nano-tweezing^26, 27^. In an imaging format, plasmons have been used to map metallic nanostructures^15, 28, 29^ or biological structures^30^ via SERS with sub-diffraction limited resolution^31^. On top of this, the ability to gather spectral information, while maintaining sub-diffraction-limited spatial resolution, would significantly extend these techniques. Of the many chemical identification techniques, SERS is particularly powerful^24^ and has been employed in the exploration of plant cells^32^, cancer stage detection^33^, living and dead bacterial cell differentiation^34^, and single-molecule detection and imaging within living cells^35^. However, providing a solution for super-resolution chemical imaging of cells or other samples of interest on extended plasmonic imaging substrates has not yet been fully investigated.

In this paper, we show that by exploiting a blinking SERS effect processed using Stochastic Optical Reconstruction Microscopy (STORM) algorithms^36^ and imaging through an optical diffraction grating, we are able to capture SERS spectral content and super-resolution spatial content at the same time. Thus, this technique is a class of “snapshot hyper-spectral imaging” wherein both spectral content and spatial content are obtained simultaneously. In this way, we demonstrate a chemical imaging strategy that combines SERS and STORM for super-resolved chemical imaging. The interactions between the sample of interest and these hotspots provide a phenomenon in which Raman light is emitted, blinking in time. SERS blinking has been attributed to single/few molecule dynamics^21, 37–39^. Generally, the molecules at the surface are thermally diffusing^39^ in and out the plasmonic hotspots and producing intermittent “blinks” on the ~second timescale that can be localized with super-resolution^31^. Through these SERS blinks, information can be collected regarding the interaction between the sample and the plasmonic substrate. STORM is then applied to stacked images of these SERS blinks. The emission in time allows blinks to be recorded, having their centroids localized, plotted, and compiled into a super-resolution reconstructed image. Similar reconstruction techniques are often applied to fluorescent emissions, but the use of plasmons can provide SERS, which is applicable to the imaging and fingerprinting of biological material since many biological compounds are Raman active due to a high degree of molecular symmetry. There is a wide range of reporting on the SERS spectra of microorganisms, wherein everything from sample preparation to growth media to the substrate can affect spectra, challenging the interpretation of data^40–43^. Furthermore, specific compounds may interact more preferentially with the silver surface^41^. In any case, it is widely accepted that SERS is a powerful tool for analyzing and detecting microorganisms^32, 40, 44–49^. SERS has previously been shown as a valuable tool in characterizing gram-positive and gram-negative bacteria^50, 51^. While bacterial discrimination has also been shown recently with tip-enhanced Raman spectroscopy (TERS), our technique does not require a complicated scanning probe^52^.

We have previously shown that SERS-STORM provides high-fidelity images of biological structures, such as collagen protein fibers^53^, and the light can be band-pass filtered to provide some rudimentary chemical information from the emitted SERS light^54^. However, the technique required a tedious manual tuning process of the band-pass filter and dozens of image acquisitions to provide any spectral information, and the resolution of a compiled SERS spectrum is limited to the band-pass width of the filter. Moreover, focusing a laser through the sample, as done in those experiments, gives rise to distortions when imaging thick samples. Here, we image through the substrate instead, using ultra-thin (10 nm) as-deposited silver films on glass coverslips. We use this technique to image and chemically differentiate various bacterial samples. As a single-celled organism, bacteria can survive in harsh environments due in part to their cell wall composition, which is commonly categorized into two main groups: gram-positive and gram-negative. Chemical differences between cell wall structures include peptidoglycan surface chemistry of gram-positive bacteria and lipopolysaccharide gram-negative surface chemistry. We show that SERS-STORM and imaging through a grating provides access to this chemical information, as well as generates a < 50 nm resolution image of the cell and its placement on the plasmonic substrate.

## Experimental

**Figure 1:**
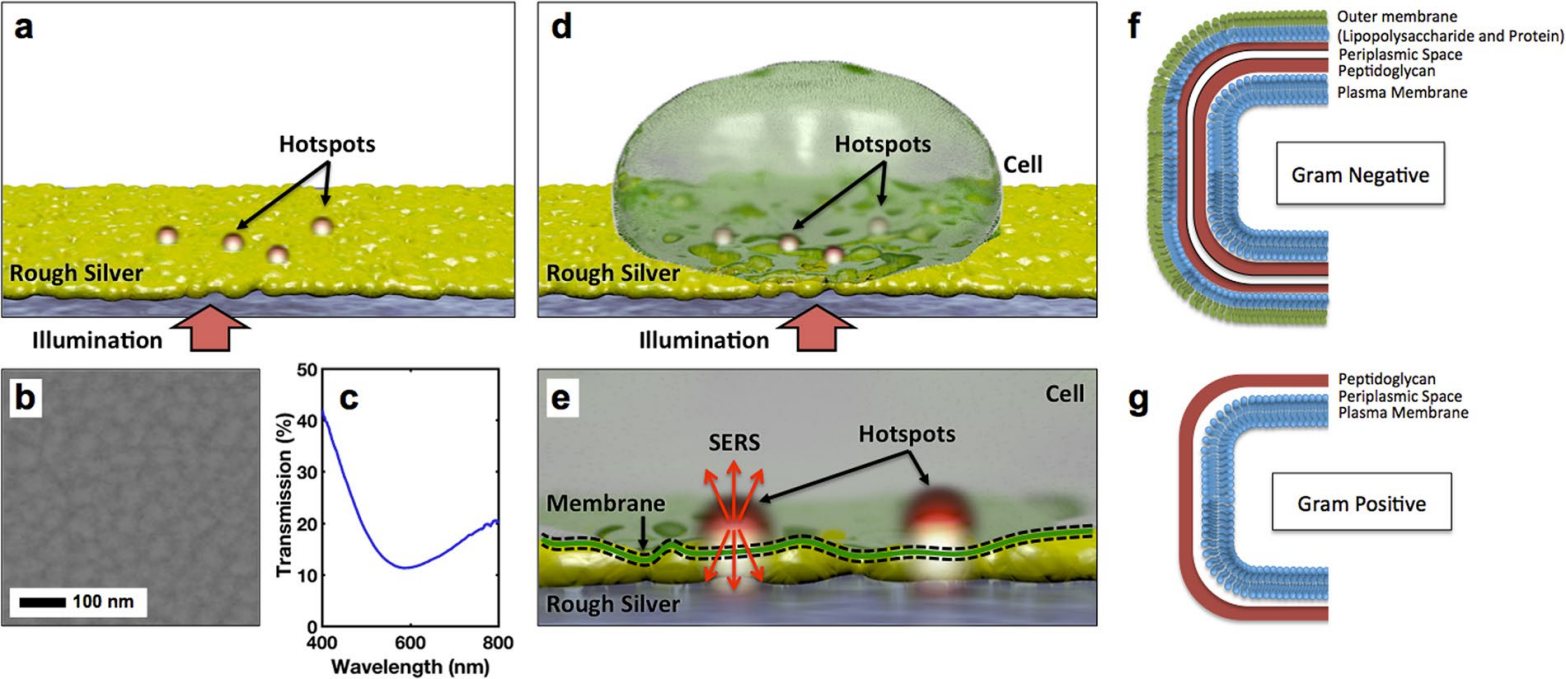
Illustration of imaging process and interaction between the plasmonic surface and the cell wall. (**a**) Depiction showing the creation of enhanced electromagnetic field “hotspots” due to illumination of the silver plasmonic surface from below. (**b**) SEM image of the plasmonic surface created by depositing 10 nm silver and a 2 nm chromium adhesion layer upon a glass microscope coverslip. (**c**) Transmission spectrum of an ultrathin silver island film showing a clear plasmonic resonance dip. (**d**) Depiction of a cell adsorbed to the rough plasmonic surface. (**e**) Schematic depicting the interaction between the hotspots and the cell wall. SERS is emitted from the sample and collected by the same objective used to illuminate from below. (**f**) Schematic depicting the molecular structure of a cell wall on a gram negative and (**g**) a gram positive bacterial species.

Plasmonic substrates were created through vacuum thermal deposition of 10 nm silver films on cleaned glass coverslips with a 2 nm chromium adhesion layer. A thin film allowed for the formation of random nano-scale features as silver condensed upon the surface into nano-islands as shown in Fig. 1a,b. The rough silver generates the plasmonic hotspots upon illumination. An optical transmission spectrum (Fig. 1c) shows a clear dip, indicating plasmonic resonances of the silver island film. While not specifically tuned to our laser wavelength of 660 nm, the dip is quite broad and provided sufficient enhancement for SERS. Chromium was used as an adhesion layer to be able to fix the cells by depositing a liquid drop and then drying. Without this adhesion layer, the 10 nm thick films could easily delaminate from the glass coverslip. Unfortunately, chromium is also an optically lossy, non-plasmonic material. Since experiments without the adhesion layer were not feasible in our current setup, it is expected that the adhesion layer has simply decreased the observed SERS signal intensity. Additional processing steps could be undertaken to optimize these substrates. For example, high-temperature annealing has been shown to increase the surface roughness of gold island films which may be beneficial for SERS^55^.

The thin plasmonic film and an oil-immersion objective allowed for imaging bacteria samples through the plasmonic surface, protecting the organism from overexposure to intense and damaging laser light while allowing plasmons to interact with surface adhesion points. This arrangement doesn’t require focusing a laser through the thick cell (which may lead to imaging distortions) and decouples the optics from the sample. While not shown here, this underside arrangement also provides the possibility of imaging the surface of adsorbed, living cells in an opaque or turbid liquid media^56^.

Three bacterial species, rod-shaped *Escherichia coli* (*E. Coli*, gram-negative), rod-shaped *Bacillus subtilis* (*B. Subtilis*, gram-positive), and spherical *Micrococcus luteus* (*M. Luteus*, gram-positive), were seeded and heat-fixed onto the plasmonic substrate. The samples were imaged within 24 hours. A bacterial colony was transferred from nutrient agar media and diluted in 2 mL of deionized water under aseptic conditions. The sample was briefly vortexed to resuspend the cells to homogeneity. Approximately 0.5 mL of the bacteria suspension was transferred to a freshly-deposited silver surface and heat-fixed for fifteen minutes at 40 deg C, rinsed, and dried using a filtered air stream. Samples were then mounted and imaged from underneath using a 100x oil objective (NA = 1.25). Upon illumination, various regions of the cell were excited via the plasmonic hotspots (Fig. 1d,e), thereby emitting a SERS signal that was collected through the same objective. This SERS light was analyzed and used to differentiate between the gram-positive and negative bacterial cell walls (Fig. 1f,g).

**Figure 2:**
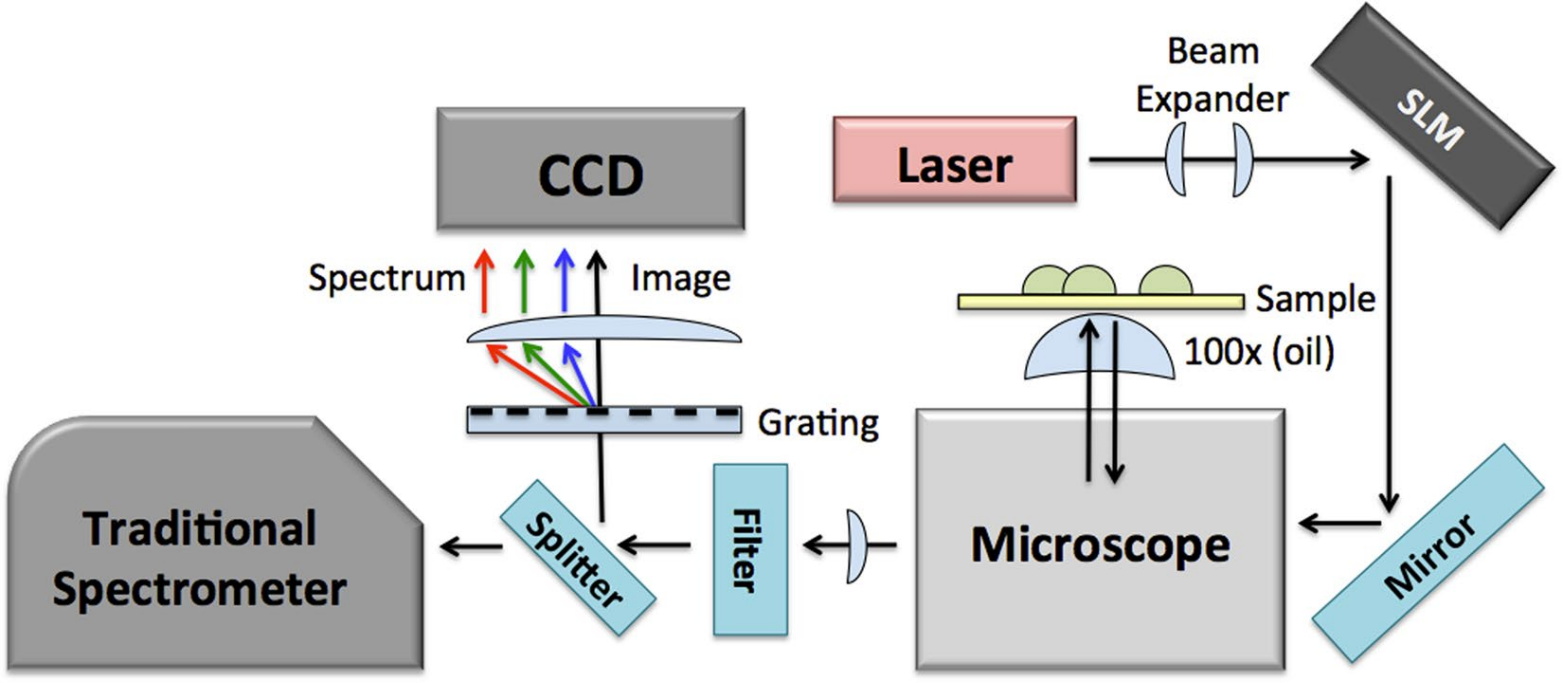
Schematic depicting the microscope and experimental setup. The light from a 660 nm laser reflects off of a spatial light modulator, allowing control and placement of the illuminating beam, and enters the inverted microscope. After interacting with the sample, the light exits the microscope and passed through a long-pass filter and a transmission grating, diffracting some of the light into a spectrum and passing the undiffracted light for imaging. The “Traditional Spectrometer” was used for traditional Raman spectral analysis and in the laser scanning Raman imaging experiments. The grating/CCD combination is a “hyper-spectral” imaging layout wherein a diffracted spectrum and a SERS-STORM image is recorded on the same video frame simultaneously on different halves of the CCD array.

Figure 2 shows the experimental setup, built around a standard inverted microscope (Nikon). The plasmonic substrates seeded with bacteria were illuminated with a 660 nm laser (Laser Quantum) covering approximately a 10 µm by 10 µm square. We have shown previously that due to the often random and non-uniform distribution of SERS hotspots, it is possible to “fill in” any gaps by randomly varying the phase of the illumination pattern^53, 54^ into a speckle pattern. This can be accomplished in several ways, including using a simple optical diffuser placed in the beam path^54^. Our current setup uses a spatial light modulator (SLM) (Hamamatsu) with a randomly varying pixel pattern. Precise spatial alignment was achieved by mounting the sample on a nanopositioning stage (Mad City Labs). Illumination power was approximately 100 mW total on the SLM and a random illumination “speckle” profile was set to change roughly every 5 to 10 seconds during image acquisition, changing the hotspot distribution across the plasmonic surface, as depicted earlier in Fig. 1a. This produced more uniform hotspots over the surface as we’ve shown earlier^54^ and allowed for wide-field SERS imaging. The changing illumination pattern was set to be slower (~10 seconds) than the time-scale of the blinks (~1 second) in order to collect many blinks for a given random illumination pattern. The collected SERS light passed first through a steep long-pass filter (Semrock) and then a 100 lines per inch transmission diffraction grating (Thorlabs). Various lenses relayed the light from the microscope through the filter and grating to the camera. Because the SERS spots were blinking in time, a movie of the surface was recorded. With an exposure time of 50 ms to 100 ms, a deep-cooled electron multiplied (EM) CCD camera (Andor) collected 3000 to 5000 frames providing a total acquisition time of a few minutes. After each imaging session, an image of two overlapping focused laser points (660 nm and 633 nm) were acquired to provide the spectral calibration. Some images were also acquired without the transmission grating for comparison. Finally, additional diffraction-limited images of the samples were acquired by scanning a focused laser over the sample with the nanopositioning stage and sending the collected light to a separate imaging spectrometer (Horiba) with a cooled CCD (Apogee). This “traditional spectrometer” setup provided reference images as well as reference spectra for comparison with our SERS-STORM images. Finally, some samples were dried and imaged directly in a scanning electron microscope (SEM) in a variable pressure, low-vacuum mode.

SERS-STORM data sets (e.g., movies of the SERS blinking effect) were processed with the open-source “rapidSTORM” software^36^. Briefly, the “rapidSTORM” software loads the movies files and analyzes them frame-by-frame for blinking events that occur above a certain threshold. These blinks, themselves the diffraction-limited point-spread-function of the imaging system, can be fit with a two-dimensional Gaussian function and the centroids can be localized to within ~10 nm. As the movie frames progress, these centroid positions and intensities are compiled and plotted in time, slowly building up the image. More information can be found in the “rapidSTORM” software manual and accompanying publication^36^. For SERS-STORM images taken with the transmission grating in place, a region of interest surrounding the zeroth order un-diffracted light was first defined. The diffracted light (the spectrum) was analyzed in custom LabVIEW^TM^ software created to correlate a diffracted spectrum to a zeroth order blink located by the “rapidSTORM” software. A super-resolution image of many of the samples was then exported and compared with SEM images of the same area.

## Results and Discussion

**Figure 3:**
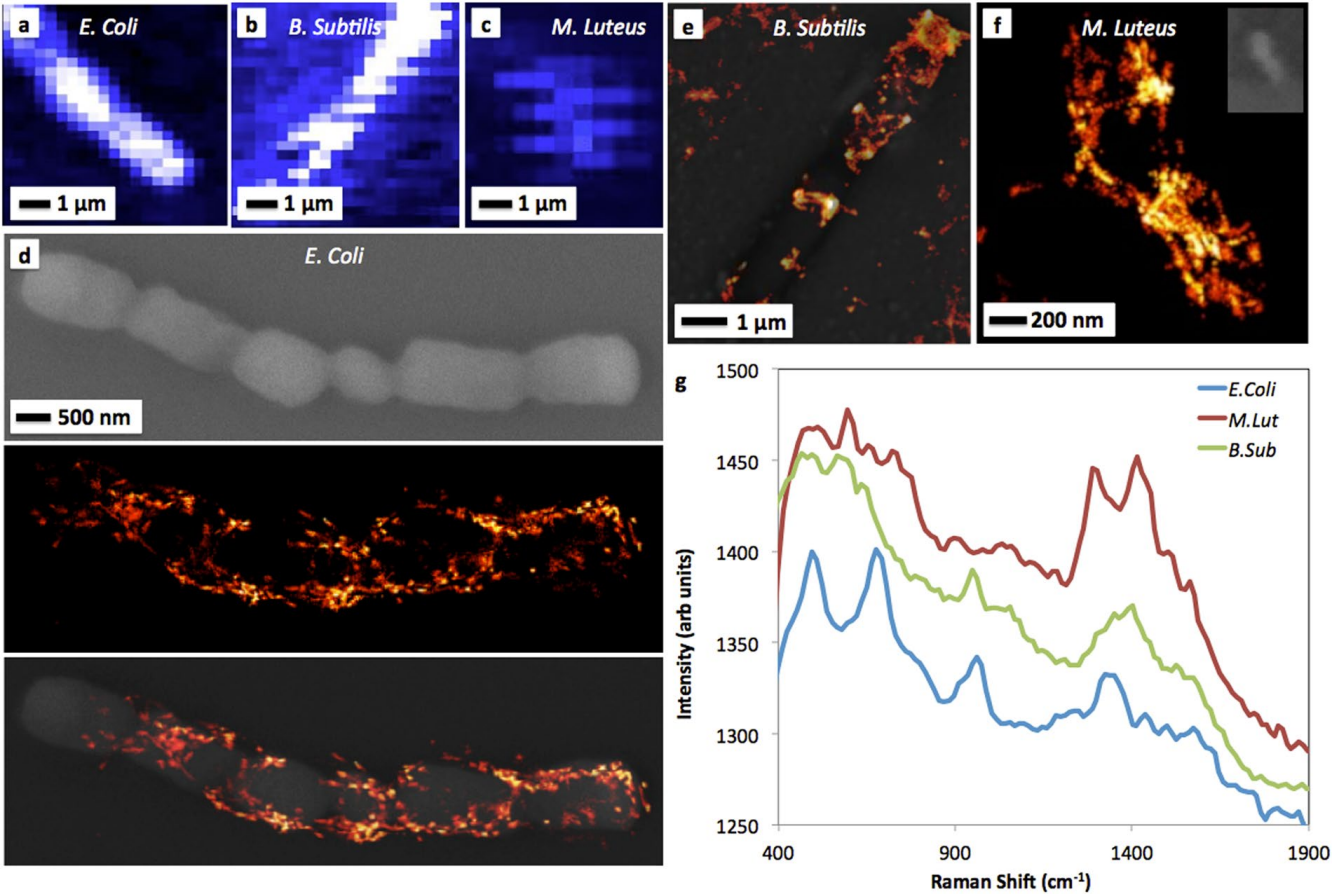
Comparison of diffraction limited laser point scans, SEM, and STORM images. (**a**) Laser scan image of *E. Coli* bacteria, (**b**) *B. Subtilis* bacteria, and (**c**) *M. Luteus* bacteria samples. (**d**) Panel of *E. Coli* images depicting (top) an SEM image of the bacteria sample, (middle) a STORM image of same sample, and (bottom) an overlay of the two. (**e**) STORM images of *B. Subtillis* overlayed with an SEM of the same region. (**f**) A STORM image of *M. Luteus* cells with an inset of an optical microscope image showing the circular shape. (**g**) SERS spectra collected from the laser points scans in panels (**a**) through (**c**) of the various bacteria types.

Bacteria species were imaged using three techniques: the proposed SERS-STORM “snapshot” imaging technique, SEM, and diffraction-limited SERS point raster scans. These imaging techniques are compared in Fig. 3. The same sample of *E. Coli* was imaged using point scan (Fig. 3a), SEM (Fig. 3d, top), and SERS-STORM (Fig. 3d, middle) techniques, showing excellent agreement between the three methods (Fig. 3d, bottom) and the morphological rod shape within the super-resolution images. Similar images were collected with *B. Subtilis* (Fig. 3b,e), where the expected rod shape is again observed. Both of the samples show significant signal from the cell membranes in the SERS-STORM images, whereas the diffraction-limited point scanning images are not able to resolve these details. We suspect that the regions of high SERS intensity are the regions of the cells that are most interactive with the silver surface. Similar imaging processes were conducted with *M. Luteus* (Fig. 3c, f), and yielded a visible spherical, coccus structure and revealing detailed 50 nm wide features of the cell wall structure. From the point scanning images taken via the traditional spectrometer, reference SERS spectra were extracted and are reported in Fig. 3g. The spectral range, however, was limited and does not extend beyond 1900 cm^−1^. Significant peaks of interest within *E. Coli* are seen around ~670 cm^−1^ (aliphatic chain vibration), ~950 cm^−1^ (C-O-C vibration), and ~1330 cm^−1^ (adenine related compounds, aliphatic chain vibrations, or CH_2_ deformation). The small peak at ~1170 cm^−1^ (tyrosine) has previously been associated with some gram-negative bacteria^50^. Those peaks corresponding to *M. Luteus* were located at ~1300 cm^−1^ (aliphatic chain vibrations or CH_2_) and ~1420 cm^−1^ (CH_2_ or CH_3_ asymmetric deformations). Wavenumber peaks of interest within *B. Subtilis* were located at ~950 cm^−1^ (C-O-C or alicyclic vibrations) and a peak again around ~1400 cm^−1^. Tentative peak assignment and identification was generally done by comparing to published data^45, 57–59^ and all compounds are those expected for a gram-positive peptidoglycan outer S-layer as well as gram-negative lipopolysaccharide and protein outer membrane^60^.

**Figure 4:**
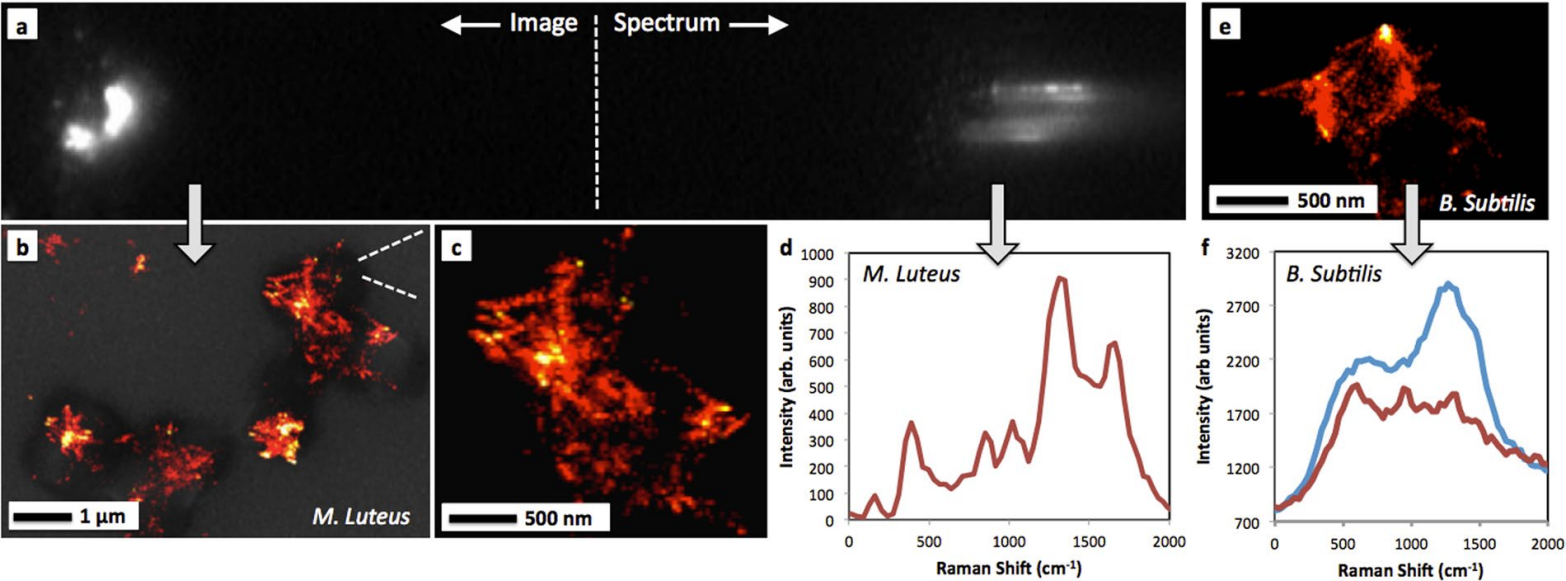
Depiction of the “snapshot” chemical imaging process by imaging through a diffraction grating. (**a**) One frame of the SERS blinking video showing the zeroth order (undiffracted) image and the diffracted spectra from each blink. This image was saturated to show both the zero order light and the weaker spectrum in the same frame. (**b**) SERS-STORM image of an *M. Luteus* sample overlaid on an SEM image of the same cells. (**c**) Blown-up region showing <50 nm details, demonstrating that the diffraction grating does not degrade the image quality. (**d**) A sample spectrum extracted from the SERS-STORM image. (**e**) Zoomed in image of a *B. Subtilis* sample. (**f**) Two spectra have been extracted from the SERS-STORM data giving peaks at similar locations to those shown by the traditional spectrometer.

Figure 4a shows a raw image frame taken with the transmission grating. The image includes the zeroth order STORM image of the cell adhesion points and the diffracted SERS spectrum. The sample shown is of *M. Luteus*. Since the spectrum is spread out over many pixels during a single short “blink” event, the signal-to-noise is typically less than with the traditional spectrometer. The relatively weaker spectral signal also required the frame to be saturated only for viewing. However, strong SERS peaks are clearly visible. Spatial information is extracted into images (Fig. 4b,c) and spectral information is extracted into a SERS spectrum (Fig. 4d). Prominent peaks include ~850 cm^−1^ (C-O-C or alicyclic vibrations), ~1020 cm^−1^ (C-O-C asymmetric vibrations), ~1300 cm^−1^ (CH_2_ or aliphatic chain vibrations), and ~1650 cm^−1^ (C = O stretch, amide I peptide band). Again, all are considered common bacterial signals^40, 61–63^ and are similar to what were shown with the “traditional spectrometer” in Fig. 3. For example, Fig. 3g and Fig. 4d both show a prominent peak at ~1300 cm^−1^. Figure 4e shows a zoomed-in image from a *B. Subtilis* sample. Two spectra were then extracted from the diffracted light from two different locations. The spectra in Fig. 4f show peaks at around ~600 cm^−1^, ~950 cm^−1^ and ~1300 cm^−1^. These are again similar to those peaks from the traditional spectrometer in Fig. 3g for *B. Subtilis*. Since SERS blinking is characterized by spectral features appearing and disappearing, it is also expected that not all of the peaks will appear with the same prominence in every spectrum^39^.

Due to a larger phospholipid lipopolysaccharide outer membrane (Fig. 1e), gram-negative bacteria (*E. Coli*) are expected to exhibit greater lipid signature within Raman spectra than gram-positive species (*M. Luteus* and *B. Subtilis*). Also, the strong peak at ~1330 cm^−1^ has been used previously to help separate *E. Coli* from other bacteria^64^. Within gram-positive species, wall teichoic acids comprise a large component of the peptidoglycan linked polymers^65, 66^. Correspondingly, both types have significant protein composition and should have signal of a peptide bond. The *M. Luteus* spectrum in Fig. 4d shows a strong amide I peptide bond at ~1650 cm^−1^ that can be associated with gram-positive species^51^. The gram-positive species include an outer S-layer high in glutamic and aspartic acid and lysine content^60^. This provides additional opportunities to explain the signals detected. Peaks at ~1300 cm^−1^, ~1400 cm^−1^, and ~1650 cm^−1^ have also been associated with the thick outer peptidoglycan layer of gram-positive bacteria^52, 59^. The *B. Subtilis* spectra in Fig. 4f show strong peaks at ~1300 cm^−1^. Finally, the S-layer includes many hydrophobic amino acids, providing for aliphatic chain vibrational signals^60^.

**Figure 5:**
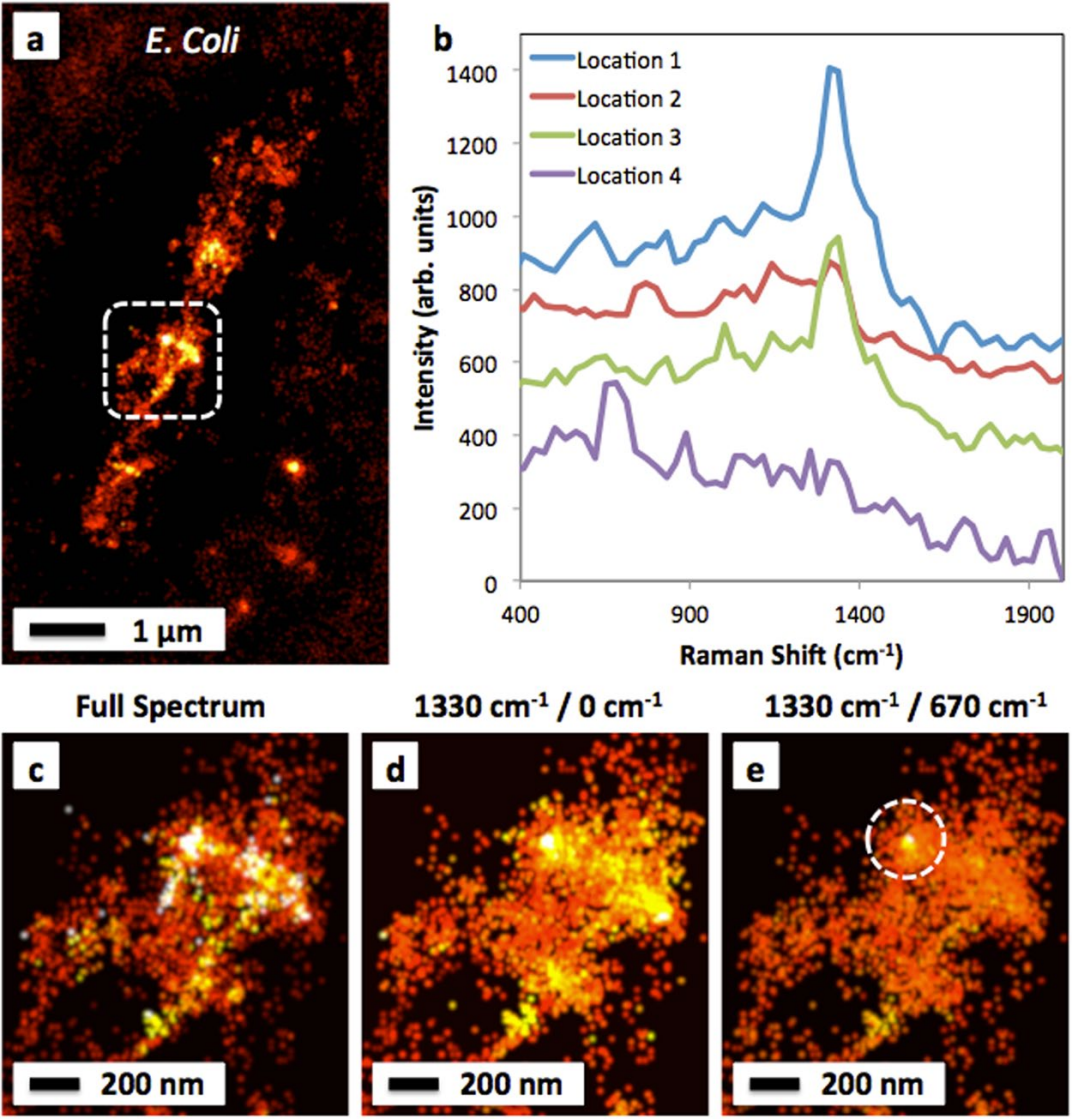
Features of the SERS-STORM data set. (**a**) An image of an *E. Coli* sample. (**b**) Several spectra have been extracted from the diffracted light on the CCD from various locations of the cell, scaled and shifted vertically to fit. (**c**) A zoomed view of the dashed region. This image has been produced from the zeroth order un-diffracted light, i.e. all of the SERS light with no spectral information. Using the spectral data shown in panel (**b**), it is possible to produce **(d)** an image of the ~1330 cm^−1^ peak intensity normalized to the residual laser line intensity at 0 cm^−1^, thus removing inhomogeneities, or (**e**) an image of the ~1330 cm^−1^ peak intensity normalized to the peak at ~670 cm^−1^. A region of interest circled in the image shows particularly intense ~1330 cm^-1^ light remaining after such normalization.

Adding the grating does not reduce image quality and produces a “hyper-spectral” data set. A super-resolved chemical image of another *E. Coli* sample is shown in Fig. 5. The spectral content of the image again matches what is gathered with the traditional spectrometer by showing a prominent feature near ~1330 cm^−1^ due to CH_2_ deformations in lipids or carbohydrates as well as several other smaller peaks near ~670 cm^−1^ and ~770 cm^−1^ from several different locations on the cell (Fig. 5b). With these datasets, it is therefore possible to produce images at different wavelength bands. For example, Fig. 5c shows a zoomed in region of the cell imaged via the full, integrated SERS signal (i.e., all the light in the non-diffracted portion of the CCD frame), as with all of the images shown previously. Alternatively, Fig. 5d shows an image that has been reconstructed exclusively from the *spectral* portion of the CCD frame. Specifically, it shows the intensity of the ~1330 cm^−1^ peak normalized (divided) by the intensity of the residual laser line at 0 cm^−1^ as a reference. This shows more homogeneity in the image since it normalizes the SERS spectral light to any variations in the laser illumination intensity, light scattering, or hotspot generation. This type of normalization would not be possible without the correlated spatial/spectral data. Finally, Fig. 5e shows the ~1330 cm^−1^ peak normalized by the spectrum at ~670 cm^−1^. This produces an image that will show areas of particularly strong 1330 cm^−1^ signal with respect to the general intensity of the rest of the SERS spectrum. While the image has again become more homogeneous, as might be expected, there remains a region of the image (circled) that corresponds to an especially intense 1330 cm^−1^ signal. In this way, successful simultaneous acquisition of both super-resolved images as well as correlated Raman spectra can be achieved.

The SERS-STORM images have limited depth of imaging (~10 nm), as the plasmonic hotspot interactions occur only at the surface interface, making this technique especially suited for imaging cell adhesion points or cell interaction with a surface. For example, the presence of the 730 cm^−1^ peaks^67^ in Fig. 5b may suggest adenine-related compounds to be present on the surface of these *E. Coli* cells^41^. Furthermore, some of the images in Fig. 3 indicate that the cell wall is outside of this plasmonic depth of field (i.e., it is dark), which suggests the cells are not resting completely flat upon the surface (e.g., Fig. 3d). This response may be due to heat fixation or to heterogeneity on the bacterial cell surface and its interaction with the surface^68, 69^. However, Fig. 3f, Fig. 4b,c,e, and Fig. 5 show more uniform contact for the three species imaged.

To conclude, this research focused upon the development of a SERS-STORM “snapshot” super-resolution chemical imaging technique. Further work and investigation in optimizing the technique could include increasing the density of the transmission grating to increase the specificity and resolution of the SERS spectra and increasing signal-to-noise by optimizing the substrates. Initial analysis shows promise for the ability to differentiate between the chemical signatures of different bacterial species while at the same time imaging their structure. For example, one of the strongest peaks at ~1650 cm^−1^ within an *M. Luteus* spectrum (Fig. 4d) corresponds to the amide I band characteristic of a polypeptide bond. The strength of the peak is promising because one would expect stronger peptide signal within the peptidoglycan coating of gram-positive species. Additionally, the peak at ~1330 cm^−1^ seen within the *E.Coli* bacteria samples would be an expected strong signal^70^ from the lipopolysaccharide of gram-negative species. While an increase in sample size would be required to make a statistically significant claim in differentiating between the various bacteria as has been shown in some previous studies^51^, our technique shows promise in this application. Furthermore, due to the relative simplicity in both the nanofabrication of the substrates and the optical imaging setup, we conclude that these results have potential as a new biological imaging technique that offers both high spatial resolution and chemical content simultaneously.
